# Curosurf surfactant application on preterm babies with respiratory complications-health-economic benefits

**DOI:** 10.4314/ahs.v24i1.27

**Published:** 2024-03

**Authors:** Anna Mihaylova, Kilova Kristina, Petya Kasnakova, Stanislav Gueorguiev, Petkova Gueorguieva, Desislava Bakova, Nikoleta Parahuleva

**Affiliations:** 1 Department of Health Care Management, Faculty of Public Health, Medical University of Plovdiv, Plovdiv, Bulgaria, 15A Vasil Aprilov str., 4002 Plovdiv, Bulgaria; 2 Department of Medical Informatics, Biostatistics and e-Learning, Faculty of Public Health, Medical University of Plovdiv, Plovdiv, Bulgaria, 15A Vasil Aprilov str., 4002 Plovdiv, Bulgaria; 3 Department of Pharmaceutical Sciences, Faculty of Pharmacy, Medical University of Plovdiv, Plovdiv, Bulgaria, 15A Vasil Aprilov str., 4002 Plovdiv, Bulgaria; 4 Department of health policy and management, Faculty of Public Health, Medical University of Sofia, 15 Akademik Ivan Evstratiev Geshov Blvd., 1431 Sofia, Bulgaria; 5 Department of Obstetrics and Gynaecology, Medical University of Plovdiv, Plovdiv, Bulgaria, 15A Vasil Aprilov str., 4002 Plovdiv, Bulgaria

**Keywords:** surfactant, CUROSURF, preterm babies, respiratory complications, health-economic benefits

## Abstract

**Background:**

The implementation of surfactant for respiratory syndrome approbates the therapy as a revolutionary method in intensive neonatal therapy and respiratory resuscitation. It is important to investigate the costs of this treatment.

**Objective:**

The aim of the study is to analyze the data by the application of the surfactant Curosurf to preterm babies with respiratory complications and describe the treatment costs, healthcare resource utilization and evaluate economic benefits of surfactant use in the treatment of neonates with respiratory distress syndrome (RDS) and hyaline-membrane disease (HDM).

**Methods:**

A retrospective survey was performed covering 167 babies based on respiratory complications due to preterm birth and the necessity to apply a surfactant therapy. A documentary method was implemented and for each patient, an individual research protocol was filled out - a questionnaire created specifically for the purposes of the study.

**Results and discussion:**

An analysis of the data from the application of CUROSURF was made and the obtained therapeutic results were compared to expenditures for the therapy, short-term therapeutic effect, benefits and consequences of the therapy of preterm newborns with respiratory complications. The application of CUROSURF to babies with RDS resulted in the realization of net savings due to the elimination of the necessity of conducting several diagnostic and therapeutic procedures as well as their duration reduction of hospital stay, thus defining its health-economic benefits.

**Conclusions:**

The models of evaluation of cost effectiveness reveal that the medicinal product is expensive but effective from the aspect of short-term therapeutic results.

## Introduction

Preterm birth is a worldwide socio-economic problem. It is the most common cause of neonatal mortality 1. Approximately 11% of all infants are born preterm, and the numbers are rising in many countries internationally 2. In neonatal medicine, more and more new and adaptive therapies are applied in premature birth, with the aim of: lowering the risk of death of premature newborns; reduce in the likelihood of developing health complications in newborns of treated women to a minimum. In the different countries of the world, whether developing or developed, there are general trends towards achieving an optimal solution to the issues related to premature newborns and their families. Respiratory insufficiency is one of the most common problems for preterm birth; it manifests as respiratory distress syndrome (RDS), a product of structurally immature lungs and pulmonary surfactant deficiency. 2,3 The therapy of respiratory complications in preterm newborns as a result of primary surfactant deficiency is based on managing this state by intratracheal application of an exogenous surfactant. In order to generate the most optimal results the medicinal product should be administered as soon as possible after the birth 4,5. The exogenous surfactant is a life-saving agent applied in case of emergency to children with severe respiratory failure because of primary or secondary lack of surfactant as stipulated by the medical neonatology standard 6. The implementation of surfactant substitution therapy for respiratory syndrome approbates the medicinal product as a revolutionary method in intensive neonatal therapy and respiratory resuscitation 7. Nevertheless, it is important to investigate the costs of this treatment. Economic benefits are a healthcare priority mostly referring to a saved life or improved preterm babies' quality of life.

Surfactant is applied usually to newborns with clinical signs of Respiratory Distress Syndrome (RDS). According to Petrou et al a surfactant therapy in case of a symptomatic RDS has not increased the costs per surviving newborn 8. Newborns with higher birth weight have a greater chance to survive. In their case the reduced costs are due to prevention of severe and chronic diseases 9. The sooner the surfactant is applied in case of newborns RDS, the greater their survival chances are 10. Surfactant application before appearance of RDS signs is recommended for newborns born before 30-week gestation 11,12. Egberts has established that the preventive surfactant treatment for newborns with body weight between 800 and 1000 g had caused additional net cost for an additional surviving newborn 13 According to Yu V, the surfactant has helped newborn babies in 24 – 26-week gestation to double their survival chances without increasing the rate of severe injuries which stayed under 10% 14. LISA (less invasive surfactant administration) to prematurely born infants in the delivery-room was associated with reductions in the need for mechanical ventilation and costs of care, but was less successful in those with initial, more severe respiratory disease. 15.

R. Ramanathan presented a summary of the most essential general conclusions concerning the selection of the adequate surfactant for RDS treatment: neonatal and other forms of the respiratory distress syndrome were the most frequent cause for respiratory failure in preterm born babies, especially those born before < 30 weeks gestation 16. Since the 70s of the 20th century continuous positive airway pressure (CPAP) has been the basic method for RDS treatment. The surfactant therapy was promoted in the 80s and became the standard treatment for children with RDS or at RDS risk. It has been confirmed that surfactant therapy reduced extra-alveolar gas collection, neonatal mortality rate and mortality rate in suckling age as well as costs for treatment of the surviving infants^17^,^18^. Natural surfactants extracted from animal sources containing surfactant proteins (SP-B) and (SP-C), as well as synthetic surfactants with functional protein analogues of SP-A and SP-C have been comprehensively studied in preterm newborns with or at risk of RDS.

Certain comparative assessments have been published presenting significant differences between three natural surfactants concerning treatment outcomes and treatment costs. Several prospective randomized controlled trials and retrospective studies on the natural surfactant preparations have revealed that treatment with Poractant alfa, compared to treatment with Beractant or Calfactant, resulted in significantly lower mortality rate, smaller additional doses, faster oxygen exhaustion and reduced hospital expenditures.

From economic effectiveness aspect of the treatment with poractant alfa and the market leader beractant, poractant alfa has been established by all surveys as less expensive than beractant. The smallest difference in the value of the two preparations was close to 20%; this difference could provide substantial economy for some health establishments. The difference depended on the analytical basis; the greatest difference was found for the single application model and average weight by the test survey of Speer G. 19, and the smallest one – by the multiple application model and precise dosing by weight by the test survey of Ramanathan R. 20.

The main factor playing a role in the observed economies with those economic models might be related to the circumstance that poractant alfa needed less additional doses than beractant. Another factor of economy with the single dose version was the greater concentration in ml for poractant alfa (120 mg/1.5 ml and 240 mg/3 ml) versus 100 mg/4 ml, 200 mg/8 ml for beractant. In this case the greatest advantage could be established with newborns needing only one flask 21. The criteria for required further doses were also a factor affecting the costs. In spite of the advantages provided by the surfactant therapy, the increased costs associated with the advanced health care for sub-populations, such as preterm newborns have provoked certain suspect concerning the economic part of the problem. The health care for this population has exhausted substantial health resources 22,23. The costs per year for hospitalization associated with health care for children with non-complicated clinical state of respiratory distress syndrome during the 90s were in the range $27 224 to $101 867 24,25. During the 10s of the new centuries the average annual expenses of USA hospitals for surfactant were about $113 000. The expenditures of some institutions with large neonatal wards and of specialized pediatric hospitals could reach $300 000. With the economic advantage of the surfactant therapy the surveys started focusing on the comparison of the pharmaco-economic assessment of various agents attempting to identify those to be included in the positive list.

Analyzing the pharmacoeconomic aspects of the application of exogenous surfactants for prophylaxis provide an opportunity to compare therapeutic results, the cost of treatment, the costs of therapy, the short-term therapeutic effect, the benefits and consequences of therapy in prematurely born children with HDM and other forms of RDS. Early pharmacoeconomic models depend on available technologies, health care costs, and drug prices. Finding the answers and solutions to the existing questions related to the prevention and treatment of RDS would lead to lowering the risk of neonatal mortality and to preventing or reducing the occurrence and development of additional complications accompanying RDS, respectively increasing the quality of life of the newborn. From a pharmacoeconomics perspective, it would result in a net cost savings per newborn life saved, which has implications for reducing the direct costs of drug therapy and reducing overall health care costs.

## Aim

The aim of the study is to analyze the data by the application of the surfactant CUROSURF to preterm babies with respiratory complications and describe the treatment costs, healthcare resource utilization and evaluate economic benefits of surfactant use in the treatment of neonates with RDS and HDM.

## Materials and methods

A retrospective and a prospective survey were performed covering 167 preterm babies based on respiratory complications due to preterm birth and the necessity to apply a surfactant therapy. They were divided into two main groups: “cases” or a study group of 89 premature children with prenatal corticosteroid prophylaxis and a second group of “controls” or a control group of 78 premature children without prenatal prophylaxis administered to mother.

For the purposes of the study, an additional intra-group regrouping is implemented based on some indicators:

- whether the prophylactic corticosteroid course has been completed, incomplete or missing.

- newborns to whom surfactant was administered, the data are analyzed/compared between children with insufflated natural surfactant and those without administration.

The working group of 89 prematurely born children was divided into 45 children with a full course of CS prophylaxis of two or three applications of dexamethasone – 2 applications amp. 6 mg/ml, total 12 mg. or 3 applications amp. 6 mg/ml, total 18 mg. And 43 children with an incomplete course of CS prophylaxis with one application of dexamethasone 6 mg/ml.

According to the postnatal complications, two groups were distinguished - premature with HMD of 25 children and those with other forms of RDS 101 children. From them, a group with applied life-saving surfactant therapy was formed of 37 children with single or repeated insufflation of exogenous surfactant CUROSURF 120 mg/1.5 ml sterile suspension.

For each patient, an individual research protocol was filled out - a questionnaire created specifically for the purposes of the study. A documentary method was implemented where the primary data concerning preterm babies were collected from the “Case Record”, “Mother's Epicrisis” and “Newborn's Epicrisis”.

The subject of monitoring were respiratory complications, clinical-laboratory results, length of hospital stay, oxygen therapy and lung ventilation, surfactant application, prenatal prophylaxis with corticosteroids, other medicinal products, number and type of additionally provided services, etc., social characteristics and pharmaco-economic therapeutic aspects of preterm newborn babies with respiratory complications. The researched short-term therapeutic effects after surfactant application were: oxygen therapy duration; duration of artificial lung ventilation (ALV); number of days of stay in an incubator; Number of antibiotics types; cost of the clinical pathway per baby in euro.

“Monitored case” was the available medical documentation (clinical evidence) of each preterm newborn which complied with the criteria for participation in the survey. “Analyzed indicators” were the characteristics of the surfactant therapy, presence or absence of hyaline-membrane disease and other forms of respiratory distress syndrome, health status and additional complication of preterm newborns, pharmaco-economic indicators of the diagnostic and therapeutic activities as well as for preterm babies – costs by clinical pathway and additional expenditures.

Statistical methods were applied, the results were processed by statistical software SPSS Ver. 22. and were considered statistically significant at significance level α=0.05, surveyed indirectly by a questionnaire method with completion of an individual protocol from the available documents, cost analysis – the method was applied at evaluation of exhausted health resources associated with the value of the clinical pathways assigned to the preterm babies and the applied surfactant.

For the purpose of surfactant therapy administered to the infants covered by the study the medicinal product CUROSURF® (poractant alfa) Intratracheal Suspension (80 mg/ml) 120 mg – 1.5 ml. x 2 fl.

## Results

In order to meet the tasks of the survey an analysis of the data from the application of the natural exogenous surfactant CUROSURF was made and the obtained therapeutic results were compared to expenditures for the therapy, short-term therapeutic effect, benefits and consequences of the therapy of preterm newborns with HMD and other forms of RDS.

The exogenous surfactant CUROSURF was administered to 37 (22.2%) of the tested 167 preterm newborns.

The data presented in Table 1 outlined that all 37 children to whom a surfactant had been applied were diagnosed with other forms of respiratory distress syndrome. Preterm babies with HMD that had been subjected to surfactant insufflation were 19 of a total of 25 with HMD (76%).

**Table 1:** Newborns with administration of exogenous surfactant CUROSURF (n=37; 22.2%)

	No n (%)	Yes n (%)
Diagnosed HMD	18 (48.6)	19 (51.4)
Diagnosed RDS	0 (0)	37 (100)
Prophylaxis with dexamethasone	12 (32.4)	25 (67.6)

The studied 167 infants belonged to two groups with and without prenatal corticosteroid prophylaxis. A substantially smaller amount of surfactant was administered to the group that was preventively treated with dexamethasone than to the group of newborns without prophylaxis. Of 89 babies with corticosteroid prophylaxis only 12 (13.5%) needed surfactant insufflation. In the control group without prophylaxis, though, surfactant therapy was administered to 25 (32%) of the preterm babies. Of all 37 preterm newborns with surfactant therapy, 25 (68%) were without corticosteroid prophylaxis and 12 (32%) had undergone prophylaxis. Those data clearly outline the preventive effect of prenatal corticosteroids. The cost of medicinal therapy with CUROSURF depends on the number of flasks insufflated to the preterm baby. (Table 2) The dose form of application to the preterm babies of this study was flasks of 120 mg/1.5 ml. The therapeutic dose of 100 – 200 mg/kg body weight was administered once or multiple times. The realized particular cost for a surfactant insufflation varied by application rate and dose regime.

**Table 2:** CUROSURF® therapy costs

Surfactant	Dose form	Therapeutic dose 100 mg/kg	Package cost in Euro	Single dose cost –1 fl.	Cost of a double dose –2 fl.	Cost of a triple dose –3 fl.
CUROSURF®	susp. inst. 120 mg – 1.5 ml x 2 fl.	277 € / 1000 gr	554 €	554 €	1108 €	1662 €
CUROSURF®	susp. inst. 240 mg – 3 ml x 2 fl.	478 € / 1000 gr	956 €	956 €	1912 €	2868 €

The single and twofold surfactant applications were prevailing and their percentage rate was approximately equal. Of the studied infants there were 17 newborns with single application of two flasks of CUROSURF 120 mg (18836 €), and the other 20 needed multifold application. Of those 20 16 experienced twofold administrations, of them 7 were insufflated with two flasks each (15512 €), and 9 babies were insufflated with three flasks of CUROSURF 120 mg (14958 €). Three-fold surfactant administration was observed only in 10% of the cases – 4 of the studied babies were treated with three-times application of 3 flasks of the exogenous surfactant. This fact was associated with the direct costs for the preterm newborns with HMD and other forms of RDS, as the algorithm of the clinical pathway involved only single and multifold surfactant application. The surfactant therapy is included in the current Neonatology medical standard. Surfactant insufflations is provided by two clinical pathways, namely Clinical Pathway (CP) 277 Diagnostics and intensive treatment of newborns with single surfactant application not depending on the body weight at a cost of 1643 € and Clinical Pathway 278 Diagnostics and intensive treatment of newborns with multiple surfactant application not depending on the body weight at a cost of 2835 €. The expenditures for babies following CP 277 are 29931 €, and for those following CP 278 - 56700 €.

**Figure 2:**
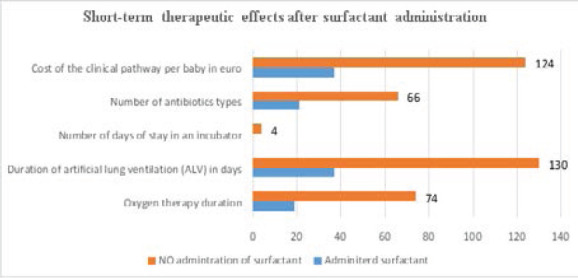
Short-term therapeutic effects after surfactant administration

The data for the indicators of short-term therapeutic results showed significantly lower values for the group with administered surfactant. The evaluation of the therapeutic effect of the applied surfactant showed benefits referring to the short-term therapeutic outcomes – reduced stay in an incubator, number of days at oxygen therapy, number of days at artificial lung ventilation (ALV), intubation, necessity of antibiotics administration, etc. (Table 3 and Fig. 2).

**Table 3:** Short-term therapeutic effects after surfactant application

Therapy / days / cost	Applied surfactant	n	Mean (SD)
Oxygen therapy duration	Yes	19	17.84 (16.344)
	No	74	6.97 (8.168)
Duration of artificial lung ventilation (ALV)	Yes	37	5.43 (11.430)
	No	130	.55 (1.633)
Number of days of stay in an incubator	Yes	0	
	No	4	7.50 (1.732)
Number of antibiotics types	Yes	21	3.86 (1.621)
	No	66	2.15 (1.026)
Cost of the clinical pathway per baby in Euro	Yes	37	1643 / 2835 (2341.378)
	No	124	160 /1590

## Discussion

According Wao et al. early surfactant administration predicted a shorter duration on mechanical ventilation (MV) and fewer patients requiring MV in the first 72 hours compared with standard surfactant administration. Similarly, early selective surfactant administration via LISA and INSURE techniques compared with standard surfactant treatment prevented some adverse long-term complications, reduced the number of deaths, and decreased length of stay and duration of MV. The proportion of patients who met the criteria to receive surfactant treatment in the alternative scenarios was higher than the base case. The estimated duration of MV and the number of patients requiring MV in the first 72 hours were reduced, and fewer patients developed bronchopulmonary dysplasia (BPD). Lower FiO2 threshold led to increased early surfactant administration, which was associated with more patients receiving surfactant, fewer patients requiring MV, shorter duration of MV, and fewer BPD patients and deaths 26.

Dani et al. demonstrated that the total cost for early surfactant administration was lower than with standard administration. They found that cost of surfactant was higher in the early surfactant administration group, but this was compensated for by lower MV expenses. Our study confirmed the same cost comparison while adding to the evidence base that the decline of excess adverse events for the early treatment group also brings down costs 27. Verder et al19 showed greater clinical efficacy with early surfactant treatment in that it reduced the overall mortality and adverse events and reduced the frequency of MV compared with standard administration 28.

The annual treatment costs of surfactant drugs in the hospital are changing, and the market prices of the units are quite high. Major problems emerge regarding both treatment and cost for the most appropriate preparation for each patient and determining the vial size, as seen in surfactant-related studies. The time between patient arrivals and the random changes in patients' weights over time complicate this problem. Treatment is applied by calculating the required amount of surfactant per the patient's weight and selecting the vial containing a sufficient amount of surfactant. Furthermore, since the surfactant in liquid form in the contents of the vials is different in milliliters in each preparation, the vials contain different amounts of surfactant material 29.

## Conclusions

The application of the exogenous surfactant CUROSURF to babies with HMD and other forms of RDS resulted in the realization of net savings due to the elimination of the necessity of conducting a number of diagnostic and therapeutic procedures as well as their duration reduction of hospital stay, thus defining its health-economic benefits.

The necessity to apply an exogenous surfactant to preterm newborns is reduced by the corticosteroid prevention in pregnancy.

The models of evaluation of cost effectiveness reveal that the medicinal product is expensive but effective from the aspect of short-term therapeutic results.

## Figures and Tables

**Figure 1:**
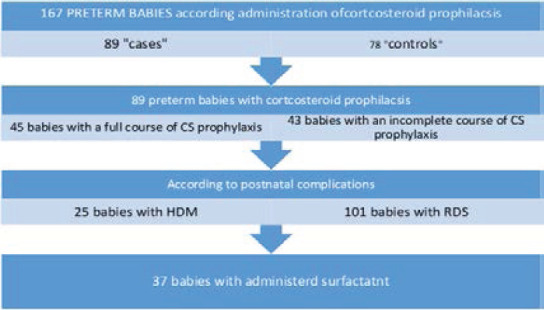
Study design

## Data Availability

The authors confirm that the data supporting the findings of this study are available within the article.
